# Genomic structure, expression, and functional characterization of checkpoint kinase 1 from *Penaeus monodon*

**DOI:** 10.1371/journal.pone.0198036

**Published:** 2018-05-24

**Authors:** Lihua Qiu, Chao Zhao, Pengfei Wang, Sigang Fan, Lulu Yan, Bobo Xie, Shigui Jiang, Shu Wang, Heizhao Lin

**Affiliations:** 1 South China Sea Fisheries Research Institute, Chinese Academy of Fishery Sciences, Key Laboratory of South China Sea Fishery Resources Exploitation and Utilization, Ministry of Agriculture, Guangzhou, China; 2 Key Laboratory of Aquatic Genomics, Ministry of Agriculture, Beijing, China; 3 South China Sea Resource Exploitation and Protection Collaborative Innovation Center, Sun Yat-Sen University, Guangzhou, China; 4 Chinese Academy of Fishery Sciences, Beijing, China; 5 Shenzhen Base of South China Sea Fisheries Research Institute, Chinese Academy of Fishery Sciences, Shenzhen, PR China; Chang Gung University, TAIWAN

## Abstract

*Chk1* is a cell-cycle regulator. *Chk1* has been identified in organisms ranging from yeast to humans, but few researchers have studied *Chk1* in shrimps. We cloned *Chk1* from the black tiger shrimp (*Penaeus monodon*). The full-length cDNA sequence of *PmChk1* had 3,334 base pairs (bp), with an open reading frame of 1,455 bp. The complete genomic sequence of *PmChk1* (11,081 bp) contained 10 exons separated by nine introns. qRT-PCR showed that *PmChk1* was highly expressed in the ovaries and gills of *P*. *monodon*. The lowest *PmChk1* expression was noted in stage III of ovarian development in *P*. *monodon*. *PmChk1* expression decreased significantly after injection of 5-hydroxytryptamine and eyestalk ablation in *P*. *monodon* ovaries. RNA interference experiments were undertaken to examine the expression of *PmChk1*, *PmCDC2*, and *PmCyclin B*. *PmChk1* knockdown in the ovaries and hepatopancreas by dsRNA-Chk1 was successful. The localization and level of *PmChk1* expression in the hepatopancreas was studied using *in situ* hybridization, which showed that data were in accordance with those of qRT-PCR. The Gonadosomatic Index of *P*. *monodon* after dsRNA-Chk1 injection was significantly higher than that after injection of dsRNA-GFP or phosphate-buffered saline. These data suggest that PmChk1 may have important roles in the ovarian maturation of *P*. *monodon*.

## Introduction

The functioning of the cell cycle involves cyclins, and leads to the division and differentiation of cells. These conversions are strictly controlled and prevent the entry of cells into the next phase of the cell cycle. The checkpoint signal plays a key role in responding to DNA damage and inducing cell-cycle inhibition [1–3]. Cell cycle checkpoint kinase 1 (Chk1) is part of the serine/threonine kinase family and is highly conserved in organisms. Chk1 was discovered first in yeast by Walworth [4, 5]. Human *Chk1* is a serine/threonine kinase comprising 476 amino acids with a molecular weight of approximately 54 kDa, and includes an N-terminal kinase domain, a variable linker region, an SQ/TQ region, and a C-terminal inhibitory structure domain [6–9]. Chk1 plays an important role in gene-expression regulation at the transition of the S phase to the G2/M phase in the cell cycle [10–12]. Thus, Chk1 is important for normal cell-cycle processes, cell differentiation, and eukaryote development [13–15].

Rb is inhibited by p21, an important target gene of p53. Rb can inhibit Chk1, which contributes to cell-cycle regulation [16, 17]. Activated Chk1 further phosphorylates the downstream protein Cdc25A/B/C to regulate the activity of maturation promoting factor (MPF) [18, 19]. MPF (cyclin B/CDC2) is a key factor in controlling mitosis. Regulation of the checkpoints at the G2/M phase requires Chk1 to inhibit MPF activity. Chk1 activity has been described in most eukaryotes [20–22]. Guo and coworkers showed that *Chk1* regulates oocyte maturation during different modes of reproduction in *Daphnia pulex* [23]. Western blot analysis has revealed that the expression of cyclin B is the highest in ovaries at stage III during ovarian development, indicating that cyclin B plays an important role in ovarian maturation in *P*. *monodon* [24]. Unilateral eyestalk ablation, which can induce spawning in *P*. *monodon* female brooders, resulted in a lower expression of cyclin A at stage IV of ovarian development [25]. In addition, a study reported that Cyclin-dependent kinase 7 gene was significantly up-regulated in stage IV ovaries in *P*. *monodon*, whereas its expression was earlier found to be high in stage III ovaries in eyestalk-ablated broodstock [26]. Evidence from the above studies showed that cell-cycle regulation is closely related to ovarian development. However, Chk1 has not been described in the black tiger shrimp (*Penaeus monodon*), and the regulatory role of Chk1 during oocyte maturation is not completely understood.

RNA interference (RNAi) has been used to observe gene function in shrimps. For instance, knockdown of *Pmp53* expression demonstrated that *Pmp53* may be important in the ovarian development of *P*. *monodon* [27]. Gonad-inhibiting hormone transcripts have been silenced using an RNAi-based method to encourage gonadal development and spawning in *Litopenaeus vannamei* [28]. Moreover, enolase is essential for the white spot syndrome viral infection in kuruma prawns [29].

*P*. *monodon* is a part of the diet for people living in south China and Southeast Asia. Eyestalk ablation causes ovarian maturation in *P*. *monodon*, but can lead to reduced egg quality and spawner mortality [30]. Hence, investigation of cell-cycle regulation can aid in the comprehension of molecular mechanisms underpinning the ovarian development and maturation of *P*. *monodon* [26]. To ascertain the molecular mechanisms of *Chk1* participation in the ovarian development of *P*. *monodon*, we first cloned *Chk1* full-length cDNA and genomic DNA from *P*. *monodon*. Next, we studied the expression of *PmChk1* transcripts in various tissues and ovarian developmental stages. This was followed by characterization of the relative expression in response to injection of dsRNA-Chk1, 5-hydroxytryptamine (5-HT), and dopamine (DA), as well as eyestalk ablation, which demonstrated the potential mechanism of *Chk1* involvement in the ovarian development of *P*. *monodon*. Among the molecules tested, 5-HT was found to stimulate the release of several crustacean hormones. It is known that the injection of 5-HT can induce ovarian maturation in crustaceans, including *P*. *monodon* [31]. The role of 5-HT in ovarian maturation of invertebrates has not been thoroughly investigated. Khotimchenko and Guerrier suggested that 5-HT may act in the optic lobe, brain, and thoracic ganglion, to stimulate gonad inhibiting hormone (GIH) or gonad stimulating hormone (GSH) secretion/synthesis, thus affecting ovarian maturation [31–33]. DA has been shown to inhibit ovarian maturation in *Litopenaeus vannamei* [34] and *Procambarus clarkii* [35], by inhibiting the release of GSH. In contrast, Chk1 may affect GSH/GIH secretion/synthesis by cell-cycle regulation, thus, indirectly affecting the ovarian maturation in crustaceans.

## Materials and methods

### Ethics statement

*P*. *monodon* is not an endangered or protected species, and there is no requirement for permission to undertake experiments involving this species in China.

### Experimental animals and preparation of samples

Experimental *P*. *monodon* (36 ± 3 g) were obtained from the Shenzhen base of the South China Sea Fisheries Research Institute (Guangdong, China). Specimens were allowed to acclimatize in aerated seawater (salinity = 30) for 3 days at 24–26 °C. Specimens at different developmental stages (spawned egg, nauplius, protozoea I–III, mysis I–III, post-larval) and ovaries at different developmental stages [I (ovogonium); II (chromatin nucleolus); III (perinucleolus); IV (yolky); V (cortical rod)] were collected from three healthy *P*. *monodon*, following which they were snap-frozen in liquid nitrogen and stored at −80 °C. Muscles, heart, hepatopancreas, ovaries, gills, brain, stomach, and intestines at stage II (chromatin nucleolus) were also collected using the same methods.

### Extraction of total RNA and synthesis of first-strand cDNA

Total RNA of dissected tissues was removed using TRIzol^™^ Reagent (Invitrogen, USA), according to manufacturer’s instructions. Total-RNA integrity was confirmed by 1.2% agarose gel electrophoresis. RNA levels were measured using a NanoDrop^™^ 2000 spectrophotometer (Thermo Fisher, USA). First-strand cDNA was synthesized from 1 μg of total RNA using a PrimeScript^™^ reverse transcriptase kit (TaKaRa Biotechnology, China).

### Cloning of full-length cDNA through rapid amplification of cDNA ends (RACE)

The partial sequence of *Chk1* was obtained from a transcriptome database. 3′ RACE polymerase chain reaction (3′ RACE PCR) was done by employing a gene-specific primer (Chk1-3GSP 1/2) and a universal primer (UPM) (Table 1). RACE PCR products were purified using a PCR purification kit (Sangon Biotech, China), ligated into the pMD18-T vector (TaKaRa Biotechnology), and sequenced (Invitrogen).

**Table 1:** Primer sequences for amplification.

Primer name	sequence (5′→3′)	application
**Chk1-F**	CCGCCTCTTTGCGCTCTAT	Cloning partial cDNA sequence
**Chk1-R**	GCGTTTCACAAGCCTCTGC	Cloning partial cDNA sequence
**Chk1-3GSP1**	AGCTTCTCGCAACCAGCCCAAC	3′RACE
**Chk1-3GSP2**	TGCCAATACCCTTGTGCCAGCG	3’RACE
**qChk1-F**	CTTGACGAGAATGACCAT	Real-time RT-PCR
**qChk1-R**	CAATACTTCAGGAGCCATA	Real-time RT-PCR
**qEF-1α-F**	AAGCCAGGTATGGTTGTCAACTTT	Real-time RT-PCR
**qEF-1α-R**	GCTTCGTGGTGCATCTCCACAGAC	Real-time RT-PCR
**iChk1-F**	CGAGCTCACGGCCTGGAAAGACCGAGATT	RNAi
**iChk1-R**	ACGCGTCGACAGGTTGGGCTGGTTGCGAGA	RNAi
**iGFP-F**	CGAGCTCTGGAGTGGTCCCAGTTCTTGTTGA	RNAi
**iGFP-R**	ACGCGTCGACGCCATTCTTTGGTTTGTCTCCCAT	RNAi
**Chk1-exF**	CGCGGATCCATGGCTGGGCCGGTCACCGA	Recombinant expression
**Chk1-exR**	CCGCTCGAGGGCTGGTAGCATATTTGTTGCTAGA	Recombinant expression
**Full-F**	ATGGCTGGGCCGGTCACCGAA	Genome full length
**Full-R**	TTAGGCTGGTAGCATATTTGTT	Genome full length
**GSP1**	CAAATTCGGTGACCGGCCCA	Genome Walking
**GSP2**	CGCCGAGCACGTGGGGCGCC	Genome Walking
**YChk1**	ATTCAATGCAGTACATGTTCCTGGAATATGCTGCAG	In situ hybridization

### Isolation of genomic DNA

Preparation of genomic DNA from muscle tissues was done following a conventional method [36]. The DNA level was estimated using a NanoDrop 2000 spectrophotometer.

### Characterization of the genomic structure and promoter region of *PmChk1*

The *PmChk1* sequence was obtained by PCR, employing genomic DNA as a template and Full-F and Full-R primers (Table 1). The expected DNA fragment was obtained using a conventional method, as described previously [37].

For determination of the 5′ upstream sequence, a Becton Dickinson GenomeWalker^™^ Universal kit (Clonetech, USA) was used, following the manufacturer’s instructions. The method used is a conventional method, and has been described previously [37]. The whole fragment of *PmChk1* was amplified by PCR using GSP1 and GSP2 primers (Table 1).

The putative promoter and transcription start site of *PmChk1* were predicted by neural network promoter prediction (www.fruitfly.org/seq_tools/promoter.html) [38]. Transcription factor binding sites were predicted using Transcription Element Search Software (TESS) (www.cbil.upenn.edu/tess) [39].

### Challenge with DA and 5-HT and the eyestalk ablation assay

For determination of the impact of 5-HT and DA on the expression of *PmChk1* mRNA, 5-HT in sterile normal saline was injected at 50 μg/g body weight into the first abdominal segment of female *P*. *monodon*; DA group was injected with DA (5 μg/g body weight) using the same method. *P*. *monodon* injected with sterilized saline solution (10 mM Tris–HCl pH 7.5, 400 mM NaCl) at 0 h acted as controls. Ovaries were harvested 0, 6, 12, 24, 48, 72, and 96 h after injection, snap-frozen in liquid nitrogen, and stored at −80 °C.

For determination of the effects of eyestalk ablation on the expression of *PmChk1* mRNA, the ovaries of *P*. *monodon* after unilateral eyestalk ablation and normal ovaries were harvested 0, 3, 6, 12, 24, 48, 72 and 96 h, snap-frozen in liquid nitrogen, and stored at −80 °C.

### Double-stranded RNAi assay

Double-stranded RNAs elicited through bacterial expression *in vivo* and transcription *in vitro* have similar silencing effects [27]. We first established the recombinant plasmids (pD7-Chk1, pD7-GFP). The primers specific to target genes (Chk1 and GFP) for dsRNA synthesis were designed and synthesized according to the gene sequences, using Primer Premier 5. Sense and anti-sense DNA templates for transcription *in vitro* were produced by employing pD7-Chk1 and pD7-GFP recombinant plasmids as templates *via* PCR. Therefore, dsRNA-Chk1 and dsRNA-GFP were synthesized *in vitro* with a Transcription T7 kit (TaKaRa Biotechnology), following manufacturer protocols. In addition, dsRNA-RBL and dsRNA-p53 were obtained from the previous experiment in our laboratory. dsRNA was stored at −80 °C.

*P*. *monodon* specimens were allowed to acclimatize for 2 days before injection of dsRNA-Chk1, dsRNA-GFP, dsRNA-RBL, dsRNA-p53, and phosphate-buffered saline (PBS). The body of each shrimp was weighted, and ovaries were dissected out from dsRNA-GFP-, dsRNA-Chk1-, and PBS-administered *P*. *monodon* and weighed. The gonadosomatic index (GSI) was calculated before the RNAi experiments. RNAi experiments were carried out using a conventional method, as described previously [27]. After injection, the ovaries and hepatopancreas of dsRNA-GFP-, dsRNA-Chk1-, dsRNA-RBL-, dsRNA-p53-, and PBS-administered *P*. *monodon* were harvested at 0, 6, 24, 48, 72, and 96 h, snap-frozen in liquid nitrogen, and stored at −80 °C. There were 10 shrimps in every group at each observation time. The GSI of each *P*. *monodon* after the RNAi experiments was calculated using the following formula [40]:

GSI=ovaryweight/bodyweight×100.

### Recombinant expression of PmChk1 and protein purification

The open reading frame (ORF) of PmChk1 was amplified using PmChk1-exF/R primers (Table 1). PCR-product cleavage was carried out with *BamH I* and *Hind III* and cloned into PET30a+ (Invitrogen). The recombinant plasmid was transformed into *Escherichia coli* BL21 (DE3)-competent cells. Fusion of PET30a-Chk1 was expressed *via* induction with 0.6 mM isopropyl β-D-thiogalactoside, upon the optical density at 600 nm (OD_600_) of the bacteria reaching 0.6. The samples were then incubated at 37 °C for 2, 4, 6, 8, and 24 h in Lysogeny Broth with 50 mg/mL ampicillin. Purification of the recombinant protein was carried out using a His GRAVI trap kit (GE Healthcare USA). The purified protein was prepared using antibodies (Jinkairui, China).

### *In situ* hybridization assay

Specific digoxigenin-labeled RNA probes against *PmChk1* were synthesized by TaKaRa Biotechnology. *In situ* hybridization experiments of hepatopancreas 24 h after injection of dsRNA-Chk1 or dsRNA-GFP were carried out using a conventional method, as described previously [41].

### Immunoblotting assay

The total proteins of ovaries after injection of dsRNA-Chk1 or dsRNA-GFP were extracted by a protein extraction kit (Kangwei, China). The proteins of ovaries, at 0, 24, and 48 h after injection of dsRNA-Chk1 or dsRNA-GFP, were separated by 12% sodium dodecyl sulfate–polyacrylamide gel electrophoresis. The gel was then moved onto nitrocellulose membranes (NMs), which were washed thrice with PBST (3% Tween 20 in PBS) and blocked with 5% skimmed milk overnight at 4 °C. A rabbit Chk1 antibody was diluted to 1:2000, and the NMs were incubated with the diluted antibody for 1 h at 37 °C. NMs were washed thrice with PBST again. NMs were incubated with diluted goat anti-rabbit antibody for 1 h at 37 °C and washed thrice with PBST again. Immunoreactive proteins were visualized following manufacturer (Tiangen, China) protocols.

### Assay to measure Chk1 kinase activity

The proteins of ovaries at 0–96 h after injection with dsRNA-Chk1 or dsRNA-GFP were detected by a tissue Chk1 kinase activity photometric assay, using a quantitative detection kit (Genmed, USA). Chk1 activity was decreased at 24 h after injection of dsRNA-Chk1, and Chk1 activity was the lowest 48 h after injection. At 24–72 h after injection of dsRNA-Chk1, Chk1 activity was significantly lower compared with the control group.

### Sequencing

The full-length cDNA sequences of *PmChk1* were assessed using the BLAST program (http://blast.ncbi.nlm.nih.gov/Blast.cgi). The complete ORF regions and amino-acid sequences were assessed using ORF finder (www.ncbi.nlm.nih.gov/orffinder/). Prediction of protein domains was done *via* SMART (http://smart.embl-heidelberg.de/). Creation of multiple sequence alignments was done using ClustalW (www.clustal.org/). Phylogenetic trees were created using the neighbor-joining method in MEGA v5.03.

### Quantitative real-time PCR

Employment of qChk1-F/R primers (Table 1) during qRT-PCR enabled estimation of the temporal expression in *P*. *monodon*. qRT-PCR was done using SYBR^®^ Premix Ex Taq^™^ II (TaKaRa Biotechnology). qRT-PCR was performed using a conventional method, as described previously [42].

### Statistical analyses

Relative mRNA expressions were assessed using one-way ANOVA via PASW v18.0 (IBM, USA) in SPSS v22.0 (IBM) and Duncan’s new multiple range test (*P* < 0.05). The data were presented as mean ± SD (standard deviation). Results were considered significant at *P* < 0.05.

## Results

### Cloning/characterization of the full-length cDNA of *Chk1*

**Fig 1:**
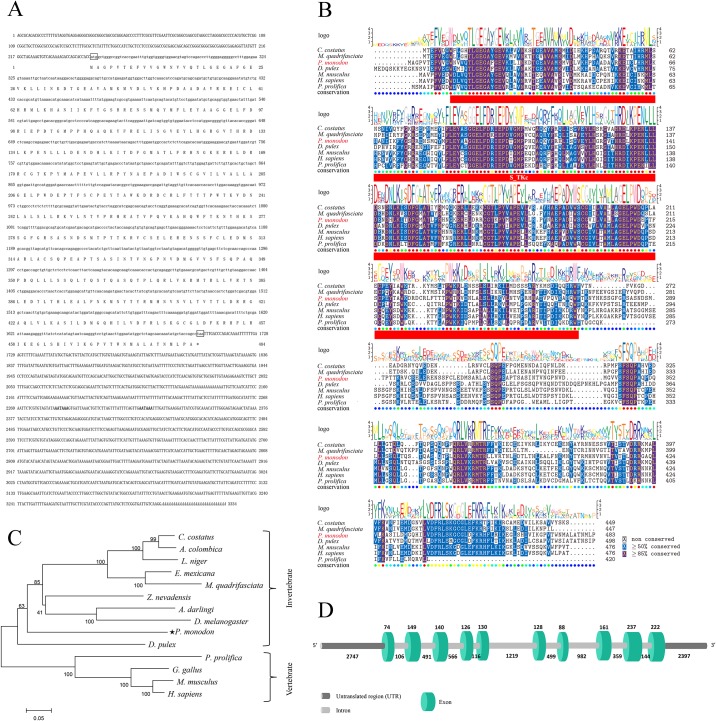
The gene information of PmChk1. (A) Full-length cDNA of *PmChk1* (schematic). The deduced amino-acid sequence is depicted underneath the nucleotide sequence. Initiation code (ATG) and termination code (TAA) are denoted by boxes. Polyadenylation signal sequence (AATAAA) is emboldened. (B) Multiple alignment of the deduced amino-acid sequences of Chk1 from *P*. *monodon* and other species. A sequence logo denoting similarity is shown at the top of alignments, and numbers of amino acids are shown on the right-hand side of alignments. GenBank numbers of Chk1 were: *P*. *monodon*: KU958380; *Homo sapiens*: AAC51736.1; *Poeciliopsis prolifica*: JAO88875.1; *Mus musculus*: AAC53334.1; *Daphnia pulex*: AGN95867.1; *Melipona quadrifasciata*: KOX69887.1; *Cyphomyr mexcostatus*: KYN05919.1. The S-TKc domain is indicated with a red box. (C) Neighbor-joining phylogenetic tree of E2F-2s based on amino-acid sequences. Confidence in each node was evaluated by 2000 bootstrap replicates using Mega v5.03. (D) Comparison of the genomic DNA sequence encoding Chk1 in *P*. *monodon*. Green-shaded rectangles denote exons, gray horizontal lines denote introns, and the numbers reflect the length (in bp) of exons and introns.

The full-length cDNA sequence of *Chk1* (GenBank accession: KU958380, S1 File) from *P*. *monodon* had 3,334 base pairs (bp), with a 5′-untranslated region (UTR) of 249 bp, a 3′ UTR of 1,630 bp, an ORF of 1,455 bp that encoded 484 amino acids, and a calculated molecular mass of 54.61 kDa. A 29-bp poly (A) tail was detected downstream of *Chk1*. The deduced amino-acid structure of *Chk1* contained a conserved S-TKc structural domain (residues 12–270) (Fig 1A).

### Phylogenetics of Chk1

Alignment of the deduced amino-acid sequences of *Chk1* genes with multiple known Chk1s is shown in Fig 1B. The functional domain of the predicted amino-acid sequence of *Chk1* from *P*. *monodon* showed the highest identity match across different species. The dendrogram in Fig 1C depicts the evolutionary relationships according to the similarity of Chk1 proteins from various species. Vertebrate Chk1 proteins were closely related and converged into a single subgroup, whereas *P*. *monodon* Chk1 proteins were clustered with other invertebrate Chk1s.

### Genomic structure and upstream regulatory region of *PmChk1*

**Fig 2:**
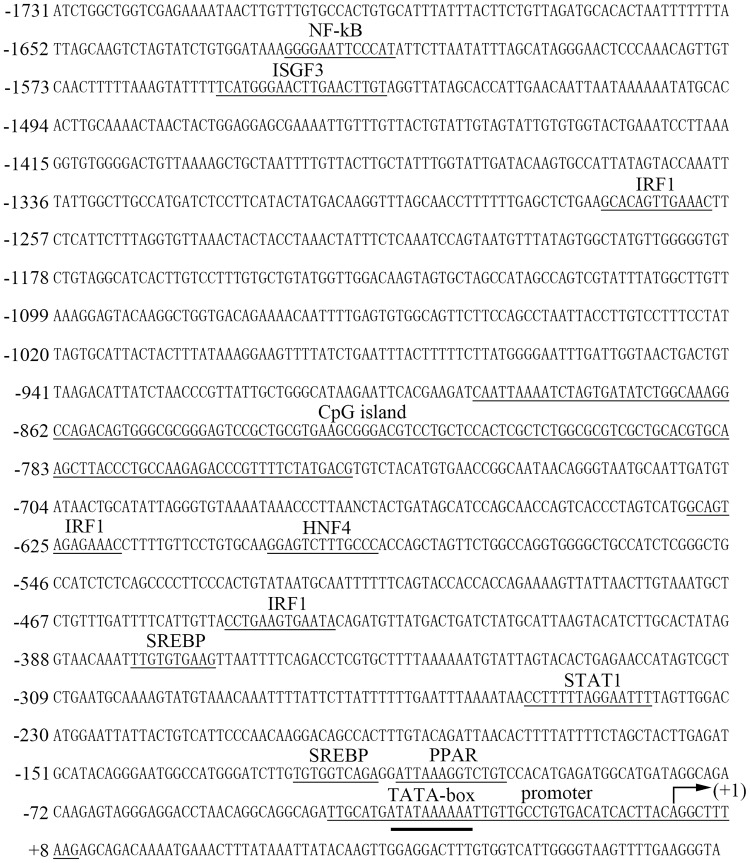
Nucleotide sequence of *PmChk1* demonstrating the 5ʹ upstream genomic sequence. Numbering of the nucleotide sequence commences from the transcription start site (+1) (arrow) and proceeds as positive numbers in the 3ʹ direction and as negative numbers in the 5ʹ direction. The putative binding sequence motifs for transcription factors and ribosomal promoter site are underlined.

We acquired the complete genomic sequence of *PmChk1* with the 5′ upstream sequence using PCR amplification of genomic DNA and genome walking. The *PmChk1* (11,081 bp) of *P*. *monodon* contained 10 exons (74, 149, 140, 126, 130, 128, 88, 161, 237, and 222 bp, respectively) separated by nine introns (106, 491, 566, 116, 1,219, 499, 982, 359, and 144 bp, respectively). This arrangement conformed to the canonical GT/AG splicing recognition rule at the extreme ends of each intron (Fig 1D). Several possible binding sites of influential transcription factors were predicted: three IRF1, two SREBP, one NF-kB, one ISGF3, one HNF4, one STAT1, one PPAR, and one TATA-box factor binding site. A CpG island was also predicted (Fig 2).

### *PmChk1* distribution in tissues

**Fig 3:**
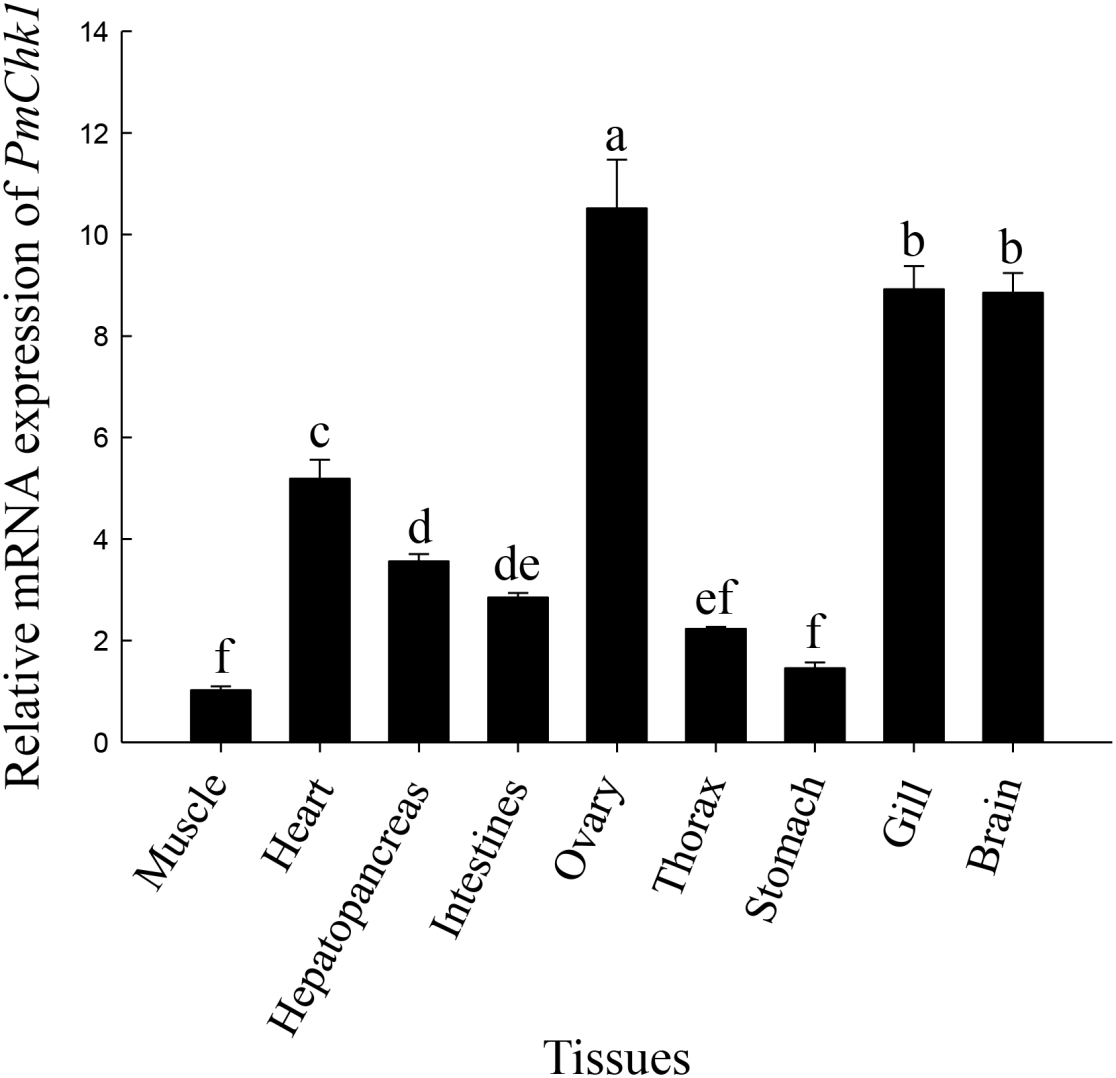
Relative expression of *PmChk1* in different tissues at stage II (chromatin nucleolus). Relative expression of *PmChk1* in different tissues according to quantitative real-time PCR using *EF-1α* as an internal reference. Vertical bars represent the mean ± SD (n = 3 for each group). Different letters above the vertical bars denote significant differences (*P* < 0.05).

The tissue-distribution patterns of *PmChk1* mRNA are shown in Fig 3. qRT-PCR data suggested that *PmChk1* was expressed widely in the muscles, heart, hepatopancreas, ovaries, gills, brain, stomach, and intestines of *P*. *monodon*. *PmChk1* expression was the highest in the ovaries.

### Expression of *PmChk1* mRNA during developmental stages

**Fig 4:**
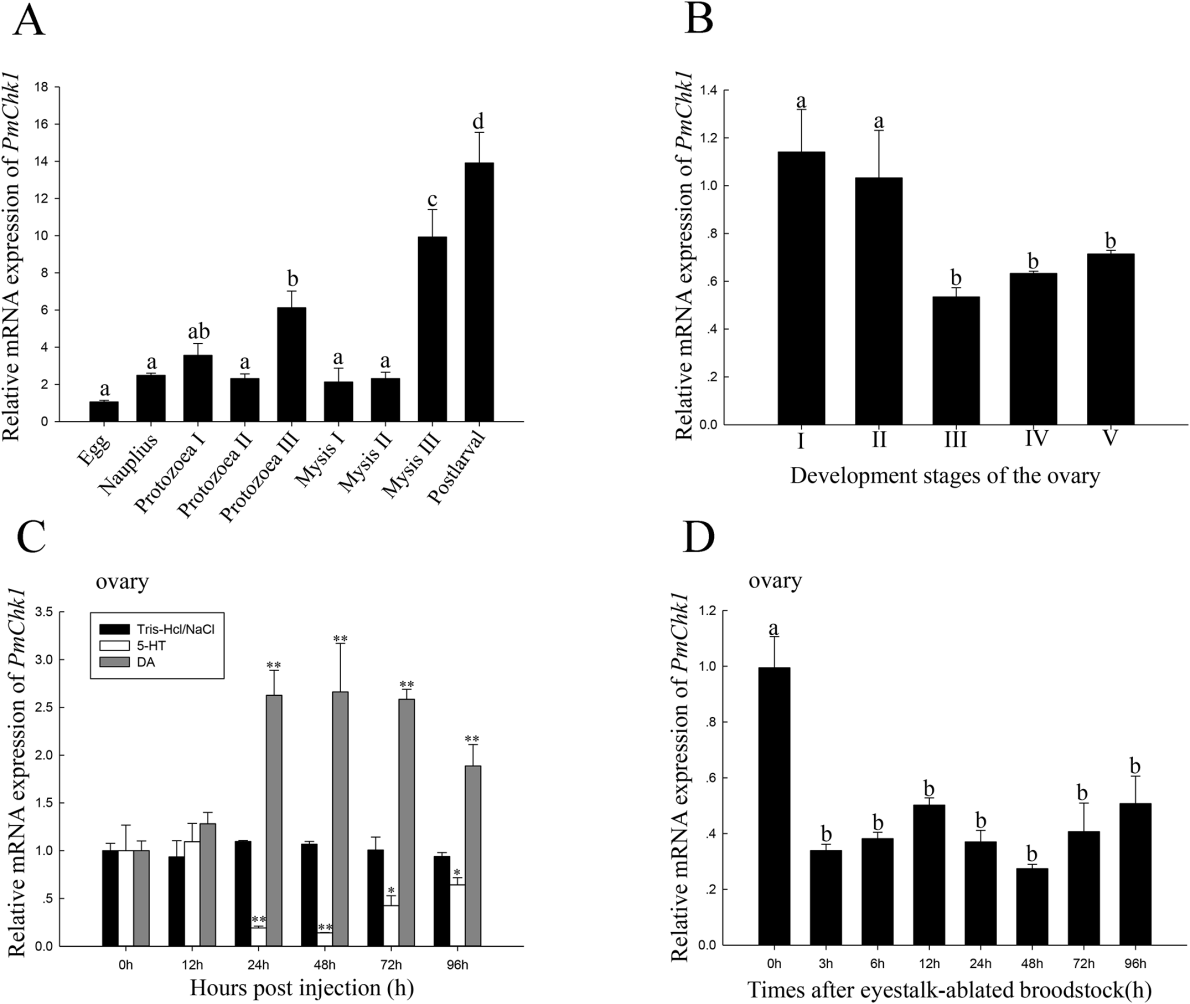
Relative expression of *PmChk1* in various conditions. (A) Relative expression of *PmChk1* in various developmental stages. The developmental stages were spawned egg, nauplius, protozoea I–III, mysis I–III, and post-larval. Relative expression of *PmChk1* in developmental stages was measured by quantitative real-time PCR with *EF-1α* employed as an internal reference. (B) Relative expression of *PmChk1* in ovaries at different developmental stages. Relative expression of *PmChk1* in ovaries at different developmental stages [I (ovogonium); II (chromatin nucleolus); III (perinucleolus); IV (yolky); V (cortical rod)] measured by qRT-PCR using *EF-1α* as an internal reference. (C) Expression of *PmChk1* mRNA after stimulation with 5-HT or DA. Relative expression of *PmChk1* mRNA in ovaries after treatment with 5-HT or DA. (D) Expression of *PmChk1* mRNA after eyestalk ablation. Relative expression of *PmChk1* mRNA in ovaries after eyestalk ablation. Vertical bars represent the mean ± SD (n = 3 for each group). Different letters above the vertical bars denote significant differences (*P* < 0.05).

The developmental-stage distributions of *PmChk1* were studied. qRT-PCR data suggested that *PmChk1* was expressed constitutively in all the developmental stages (spawned egg, nauplius, protozoea I–III, mysis I-III, post-larval) of healthy *P*. *monodon*. The highest mRNA expression of *PmChk1* was documented in the post-larval stage, followed by mysis III (Fig 4A).

### Expression of *PmChk1* mRNA during ovarian maturation

Relative mRNA expression of *PmChk1* in different ovarian developmental stages of *P*. *monodon* was detected by qRT-PCR. mRNA expression of *PmChk1* in ovarian stages III, IV, and V was significantly lower than those in I and II (*P* < 0.05) (Fig 4B).

### Expression of *PmChk1* mRNA after challenge with 5-HT and DA and eyestalk ablation

*PmChk1* expression after injection of 5-HT or DA in *P*. *monodon* ovaries was assessed. *PmChk1* expression after injection of 5-HT was significantly decreased at 24, 48, 72, and 96 h compared with the control group. *PmChk1* expression after DA injection was significantly increased at 24, 48, 72 and 96 h compared with the control group (Fig 4C). *PmChk1* expression after eyestalk ablation in *P*. *monodon* ovaries was measured. *PmChk1* expression decreased significantly at 3, 6, 12, 24, 48, 72, and 96 h compared with that at 0 h (Fig 4D).

### Expression of *PmChk1* mRNA after treatment with dsRNA-Chk1

**Fig 5:**
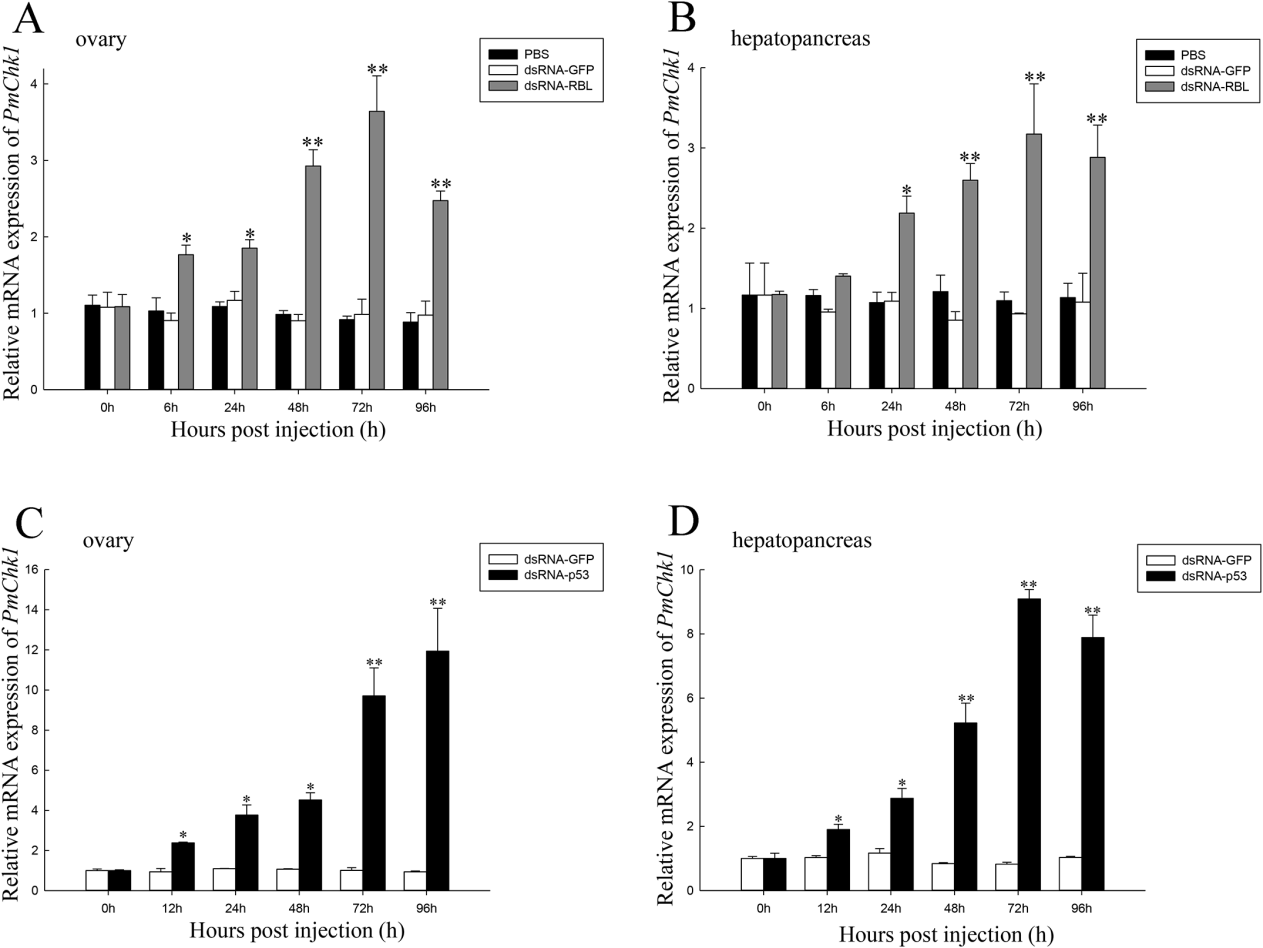
Relative expression of *PmChk1* in the ovaries and hepatopancreas of *P*. *monodon* after dsRNA-RBL and dsRNA-p53 treatment. (A) Relative expression of *PmChk1* in ovaries. (B) Relative expression of *PmChk1* in the hepatopancreas. (C) Relative expression of *PmChk1* in ovaries. (D) Relative expression of *PmChk1* in the hepatopancreas. Ovary and hepatopancreas tissues harvested from *P*. *monodon* injected with dsRNA-RBL were compared with respect to expression of *PmChk1* mRNA (relative to EF-1α) using the Student’s *t*-test. Vertical bars represent the mean ± SD (n = 3 for each group). Significant differences from controls are denoted: **P < 0.01, *P < 0.05.

*PmChk1* expression after injection of dsRNA-RBL or dsRNA-p53 in the ovaries and hepatopancreas of *P*. *monodon* was assessed. Compared with the control group, the *PmChk1* expression in ovaries and hepatopancreas was upregulated in dsRNA-RBL-injected *P*. *monodon*, from 24 h to 96 h. However, expression of *PmChk1* mRNA did not change after dsRNA-GFP administration (Fig 5A and 5B). Compared with the control group, the *PmChk1* expression in ovaries and hepatopancreas was upregulated in dsRNA-p53-injected *P*. *monodon* after 12–96 h (Fig 5C and 5D).

**Fig 6:**
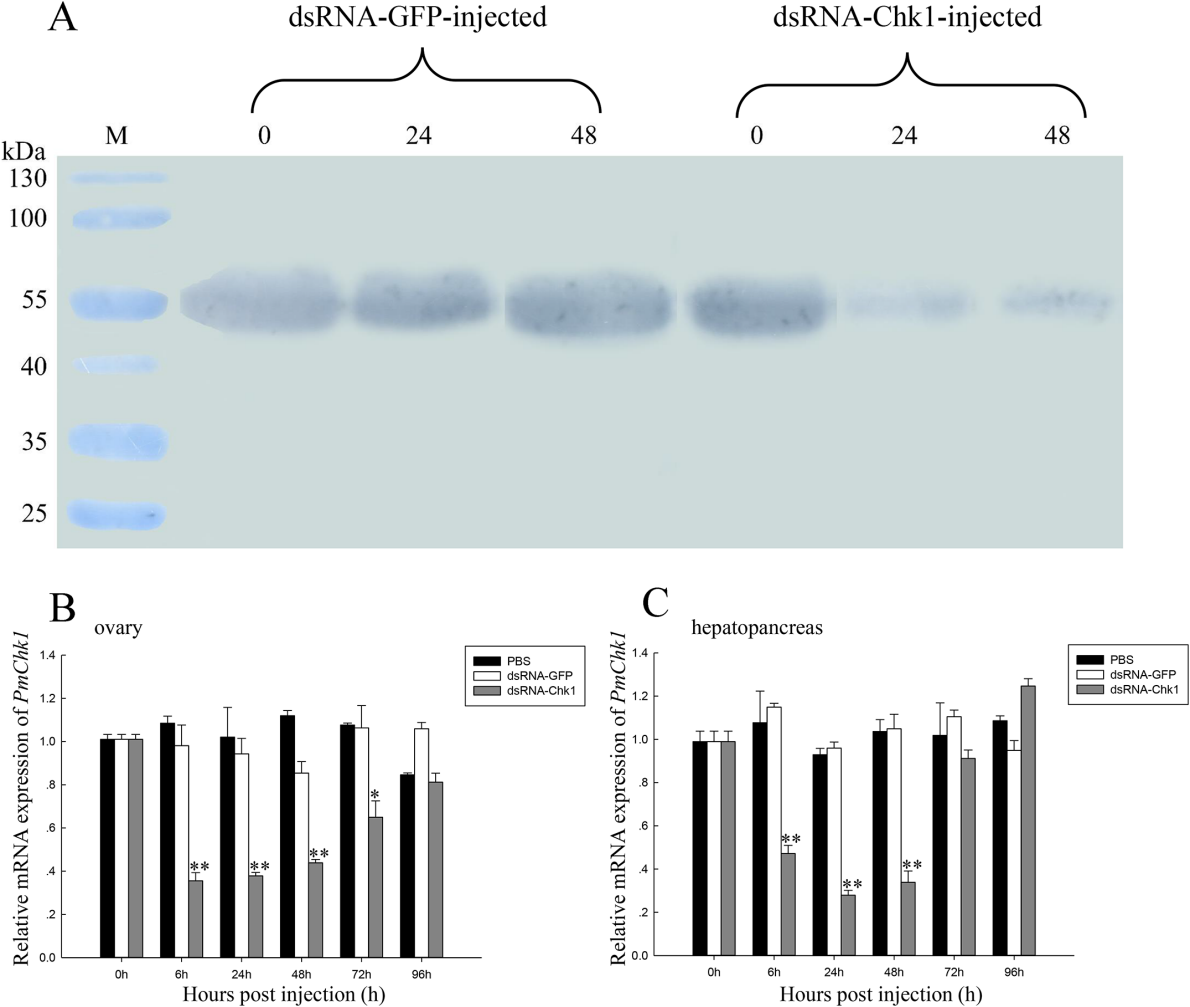
Expression of PmChk1 protein and gene after dsRNA-Chk1 injection. (A) Expression of PmChk1 protein in ovaries after dsRNA-Chk1 injection. Lane M = pre-stained molecular-weight marker. (B) Relative expression of *PmChk1* in the ovaries of *P*. *monodon* after dsRNA-Chk1 treatment. (C) Relative expression of *PmChk1* in the hepatopancreas of *P*. *monodon* after dsRNA-Chk1 treatment. Vertical bars represent the mean ± SD (n = 3 for each group). Significant differences from controls are denoted: **P < 0.01, *P < 0.05.

To determine the effects of dsRNA-Chk1 on *PmChk1* expression, we measured the expression level in the ovaries and hepatopancreas of *P*. *monodon* by qRT-PCR. *PmChk1* expression in the ovaries and hepatopancreas decreased significantly 6 h after injection of dsRNA-Chk1. *PmChk1* expression in the ovaries and hepatopancreas was inhibited in dsRNA-Chk1-administered *P*. *monodon* during 6–48 h, and then increased to levels that were not significantly different from those in the control group. In comparison, mRNA expression of *PmChk1* did not change after the injection of dsRNA-GFP and PBS (Fig 6B and 6C).

**Fig 7:**
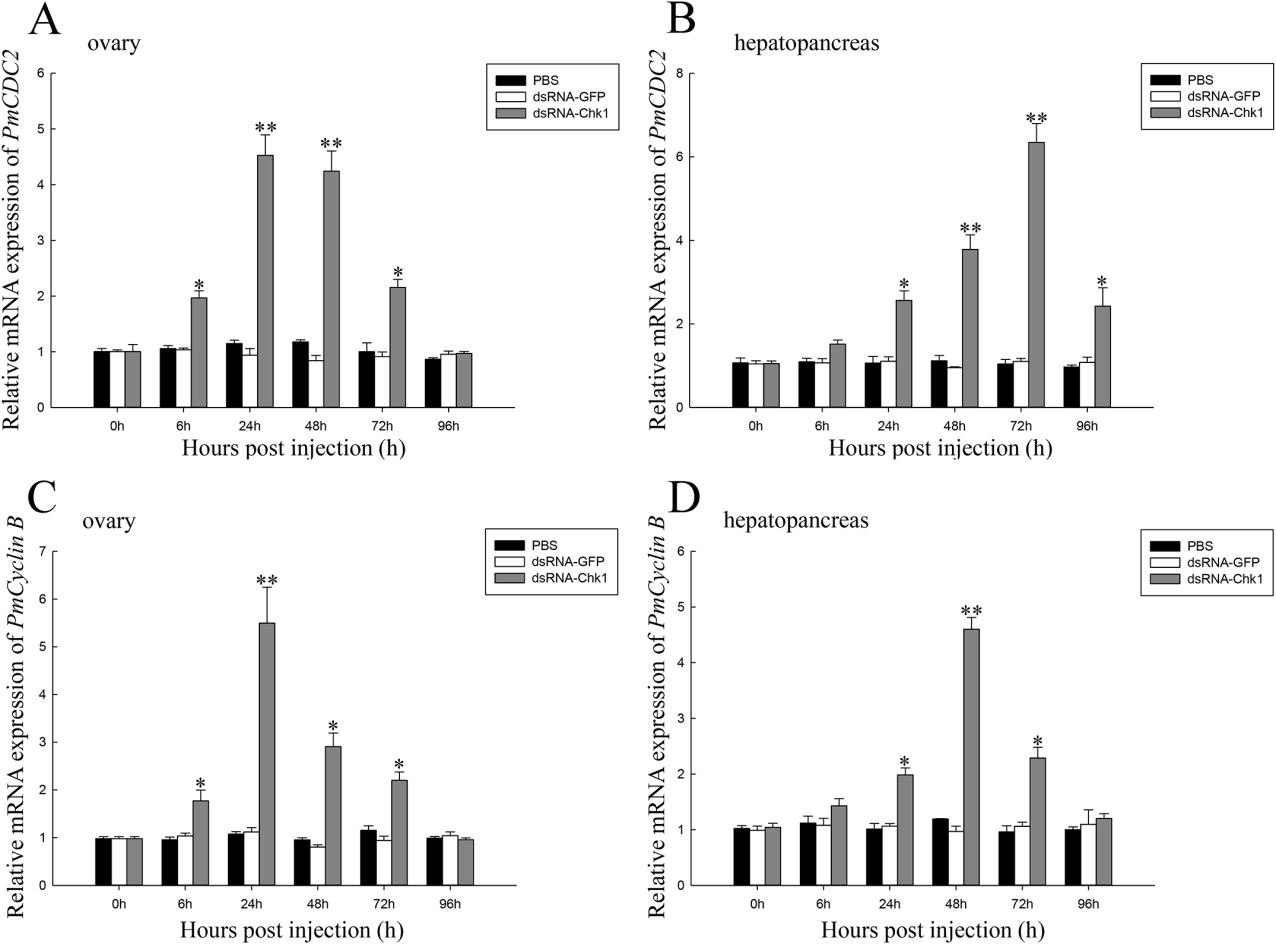
Relative expression of *PmCDC2* and *PmCyclin B* in the ovaries and hepatopancreas of *P*. *monodon* after dsRNA-Chk1 treatment. **(A)** Relative expression of *PmCDC2* in ovaries. (B) Relative expression of *PmCDC2* in the hepatopancreas. (C) Relative expression of *PmCyclin B* in ovaries. (D) Relative expression level of *PmCyclin B* in the hepatopancreas. Vertical bars represent the mean ± SD (n = 3 for each group). Significant differences from controls are denoted: **P < 0.01, *P < 0.05.

Expression of *PmCDC2* and *PmCyclin B* after injection of dsRNA-Chk1 in the ovaries and hepatopancreas of *P*. *monodon* was also studied. *PmCDC2* expression in the ovaries was upregulated 6 h after injection, and then decreased to levels similar to those in the control group after 96 h (Fig 7A). Compared with the control group, *PmCDC2* expression in the hepatopancreas was upregulated significantly at 24–96 h after injection (Fig 7B). *PmCyclin B* expression in the ovaries was upregulated 6 h after injection, and after 96 h, decreased to levels that were not significantly different to those in the control group (Fig 7C). *PmCyclin B* expression in the hepatopancreas was upregulated 24 h after injection and decreased to levels similar to those in the control group after 96 h (Fig 7D).

**Fig 8:**
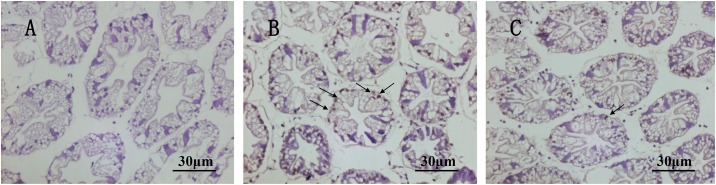
*In situ* detection of PmChk1 hybridization. The hepatopancreas was harvested after dsRNA-Chk1 administration. Brown dots denote positive reactions (arrows). A represents a negative control. B represents the collected hepatopancreas after dsRNA-GFP injection. C represents the collected hepatopancreas after dsRNA-Chk1 injection. Scale bar = 30.

To further verify the results of the RNAi assays, we carried out an *in situ* hybridization assay in the hepatopancreas to investigate the localization and mRNA expression of *PmChk1*. Positive signals were detected in the tissues after injection of dsRNA-GFP (Fig 8B), but few positive signals were observed after injection of dsRNA-Chk1 (Fig 8C). Positive signals were absent in the negative control (Fig 8A). The intensity of the positive signals indicated that *PmChk1* expression in the hepatopancreas decreased significantly after injection of dsRNA-Chk1, compared with the expression after injection of dsRNA-GFP, and the results were consistent with the qRT-PCR data.

**Fig 9:**
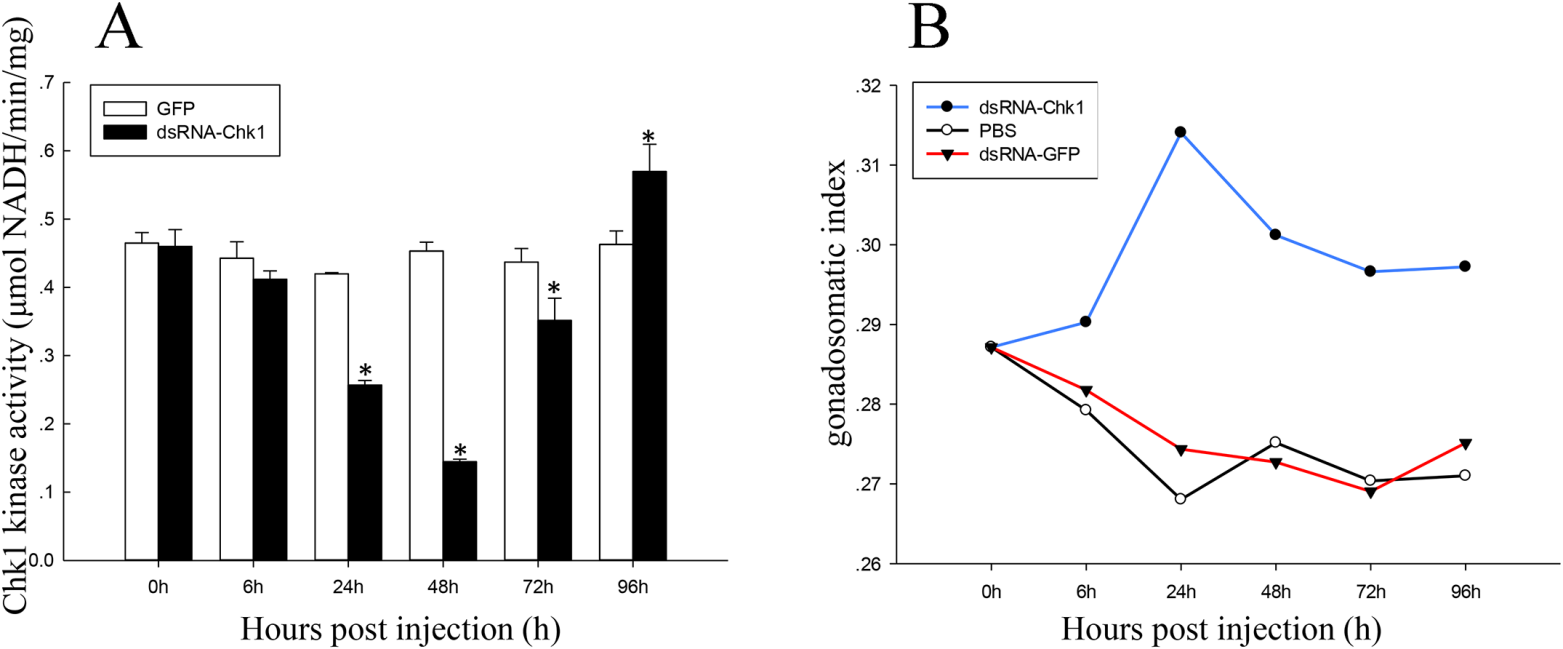
Chk1 activity and the GSI of *P*. *monodon* after dsRNA-Chk1 injection. (A) Chk1 activity after injection of dsRNA-Chk1 and dsRNA-GFP. Vertical bars represent the mean ± SD (n = 3 for each group). Significant differences from controls are denoted: *P < 0.05. (B) The GSI (ovary weight/body weight × 100) of *P*. *monodon* after injection of dsRNA-GFP, dsRNA-Chk1, or PBS.

We also examined the expression of PmChk1 protein after dsRNA-Chk1 injection using immunoblotting assays. Expression of Chk1 protein at 24 and 48 h after dsRNA-Chk1 injection was significantly lower than that that after dsRNA-GFP injection (Fig 6A). We investigated the activity of Chk1 after dsRNA-Chk1 injection by Chk1 activity assays. Chk1 expression was decreased at 24 h after dsRNA-Chk1 injection, and Chk1 activity was the lowest after 48 h. After injection of dsRNA-Chk1, Chk1 activity showed a significantly lower value compared with the control group at 24–72 h (Fig 9A).

### GSI assay

To ascertain the impact of *PmChk1* on ovarian development, the ovary weight and body weight of *P*. *monodon* were determined. The GSI of each *P*. *monodon* after injection of dsRNA-GFP, dsRNA-Chk1, or PBS was calculated. The GSI of *P*. *monodon* after dsRNA-Chk1 injection was significantly higher than that after injection of dsRNA-GFP or PBS (Fig 9).

## Discussion

Studies on Chk1 have concentrated on its role in cell-cycle regulation, including the detection of DNA damage and inhibition of tumor-cell growth [43, 44]. Researchers have identified *Chk1* in organisms ranging from yeast to humans, but few studies have investigated *Chk1* from *P*. *monodon*. Here, we first reported the cloning of *Chk1* homolog from *P*. *monodon*. The full-length cDNA sequence of *Chk1* from *P*. *monodon* was 3,334 bp long. Vertebrate Chk1 proteins were closely related and converged into a single subgroup, whereas *P*. *monodon* Chk1 proteins were clustered with other invertebrate Chk1s. The S-TKc structural domain was conserved in all selected species, suggesting that the primary structure of Chk1 proteins has been conserved during evolution.

The *PmChk1* of *P*. *monodon* contained 10 exons separated by nine introns. The 5′ upstream sequence of *PmChk1* contained IRF1, SREBP, NF-kB, ISGF3, HNF4, STAT1, PPAR, and TATA-box factor binding sites, as well as a CpG island. How these regulatory elements control the transcription of *Chk1* merits additional research.

qRT-PCR results suggested that *PmChk1* was expressed constitutively in the tissues of healthy *P*. *monodon*. The highest mRNA expression of *PmChk1* was documented in the ovaries, followed by the gills. Vitellogenin, which is derived from the ovaries, hepatopancreas, and adipose tissues in decapod crustaceans, has a crucial role in ovarian development [45]. The initiation of oocyte meiosis and maturation occurs with the termination of vitellogenesis [46]. Chk1 is not only crucial for DNA replication checkpoint and DNA damage response, but also plays an important role in the G2/M stage of mitosis [47]. Therefore, we inferred that Chk1 can affect ovarian development through regulation of cell cycle progression. Moreover, different ovarian development stages have specific effects on vitellogenesis. Thus, Chk1 indirectly controls vitellogenesis via the mechanisms of cell cycle and ovarian development.

qRT-PCR results suggested that *PmChk1* was also constitutively expressed in the developmental stages (spawned egg, nauplius, protozoea I–III, mysis I–III, post-larval) of healthy *P*. *monodon*. The highest mRNA expression of *PmChk1* was detected in the post-larval stage, and then in mysis III. Thus, *PmChk1* may play different roles in the various developmental stages of *P*. *monodon*. This hypothesis is consistent with the results of Chk1-deficient (Chk1^−^/^−^) mutant mice [48]. In nuclei, Chk1^−^/^−^ embryos showed gross morphologic abnormalities at the blastocyst stage. Moreover, Chk1^−^/^−^ blastocysts were found to have a severe defect in outgrowth of the inner cell mass and died of apoptosis [48]. *PmChk1* might function as a cell cycle regulator in oocyte maturation.

During oocyte development, various genes are expressed temporally and spatially to enable appropriate development or to retain transcripts and proteins as maternal factors for early embryogenesis [49, 50]. Stage III is an extremely important part of ovarian development, during which, oocytes that have accumulated yolk substances within cytoplasm can be observed [51]. *PmChk1* expression was the lowest in stage III, thereby suggesting an important role for *PmChk1* in ovarian development. The result is not in accordance with some studies showing that *PmCyclin B* expression is the highest in stage III [24]. These results suggest that *PmChk1* may have a negative role in ovarian development in *P*. *monodon*.

Studies of 5-HT and DA in crustaceans have focused predominantly on ovarian development. 5-HT and DA have been reported to induce/inhibit ovarian maturation and spawning in *P*. *monodon*, respectively [31, 52]. Compared with the control group, the *PmChk1* expression was decreased significantly at 24–96 h after 5-HT injection, whereas it increased during 24–96 h after DA injection. The result is in contrast to a study in which *PmCDK7* expression was enhanced after 5-HT injection in *P*. *monodon* [26]. Regulatory mechanisms of 5-HT and DA in crustaceans are not clear and merit additional investigation.

In crustaceans, eyestalk ablation is a common procedure performed to induce ovarian maturation [53]. We found *PmChk1* expression to be decreased significantly after eyestalk ablation. This result is in contrast to a study in which expression of *CDC2* in *P*. *monodon* [26] and vitellogenin in *Macrobrachium nipponense* [54] increased after eyestalk ablation. These data show that *PmChk1* may have a negative role in ovarian development in *P*. *monodon*.

Studies have described the use of RNAi to elucidate gene function in shrimps. Dai and colleagues stated that *Pmp53* knockdown had a strong impact on ovarian development in *P*. *monodon* [27]. Silencing of gonad-inhibiting hormone transcripts using an RNAi-based method can stimulate gonadal development in *L*. *vannamei* [28]. We knocked down *PmChk1* in the ovaries and hepatopancreas by injection of dsRNA-Chk1. *PmChk1* expression was inhibited 6–48 h after injection and after 72 h, increased to levels that had no significant difference compared with those in the control group. This result is in accordance with a report that demonstrated specific inhibition of gene expression by RNAi-based methods to be temporary [55].

Several scholars have focused on the use of RNAi methods to study Chk1 function. Kalogeropoulos et al. used RNAi methods to show that Chk1 is required for S-M detection during early embryonic development in mice [56]. Approximately 6–72 h after induction of dsRNA-Chk1 infection, the relative expression of *PmCDC2* and *PmCyclin B* was upregulated in the ovaries and hepatopancreas, suggesting that silencing of *PmChk1* could increase the expression of *PmCDC2* and *PmCyclin B* in *P*. *monodon*. Studies have shown that Chk1 can inhibit expression of cyclin B and CDC2, which help regulate the cell cycle. Rb is inhibited by p21, an important target gene of p53. Rb can inhibit Chk1, which contributes to cell-cycle regulation. We determined the *PmChk1* expression after injection of dsRNA-RBL or dsRNA-p53 in the ovaries and hepatopancreas of *P*. *monodon*. We found that, after 6–96 h, *PmChk1* expression in the ovaries and hepatopancreas was upregulated compared with the control group. Studies have demonstrated that *PmCdc2* and *PmCyclin B* genes/proteins have important roles in the development/maturation of oocytes/ovaries in *P*. *monodon* [24, 26]. Such data suggest that *PmChk1* may be involved in ovarian maturation.

We investigated the localization and expression of *PmChk1* in the hepatopancreas using *in situ* hybridization. Several positive signals were documented in the hepatopancreas after dsRNA-GFP injection, but few positive signals were documented after dsRNA-Chk1 injection. The quantities of these positive signals indicated that *PmChk1* expression in the hepatopancreas was decreased significantly after dsRNA-Chk1 injection, relative to the expression after dsRNA-GFP injection, and the results were in accordance with qRT-PCR data. Similar findings of RNA whole-mount *in situ* hybridization of *Chk1* in *D*. *pulex* have been reported by Guo and colleagues [23]. Immunoblotting data demonstrated that the expression of Chk1 protein at 24 and 48 h after dsRNA-Chk1 injection was significantly lower than that after dsRNA-GFP administration. Such observations suggested that the *Chk1*-silencing experiments were successful at mRNA and protein levels.

We also detected the activities of Chk1 after injection of dsRNA-Chk1 and dsRNA-GFP. Chk1 activity decreased 24 h after dsRNA-Chk1 injection, and was the lowest at 48 h after injection. After injection of dsRNA-Chk1, Chk1 activity was significantly lower compared with the control group at 24–72 h. This corresponded with the results of mRNA and protein expression of Chk1 after injection of dsRNA-Chk1 and dsRNA-GFP. Inhibitive effects of RNA interference on Chk1 gene expression caused the expression of Chk1 protein reducing, which could finally lead to the decline of Chk1 activity per unit weight of total protein.

The GSI is a gross quantitative indicator of gonad status in crustaceans. The GSI is the least complicated way to measure alterations in the size and weight of the gonads in relation to the body weight [40]. The GSI of *P*. *monodon* with dsRNA-Chk1 injection was significantly higher than that with dsRNA-GFP injection or PBS. In addition, the relative mRNA expression of *PmChk1* was significantly up-regulated after DA injection, and was significantly down-regulated after 5-HT injection. This means that 5-HT and DA can both inhibit/promote the expression of *PmChk1*, and induce/inhibit ovarian maturation in *P*. *monodon*. Evidently, injection of 5-HT and dsRNA-Chk1 could achieve the same effects in *P*. *monodon*, and *PmChk1* may have a negative role in ovarian development.

## Conclusions

cDNA sequences of *PmChk1* were cloned and identified. The complete genomic sequence of *PmChk1* contained 10 exons separated by nine introns. *PmChk1* was highly expressed in the ovary, post-larval stages, as well as in stage I–II of the ovarian development of *P*. *monodon*. *PmChk1* expression decreased significantly after 5-HT injection and eyestalk ablation in the ovaries of *P*. *monodon*. Expression of *PmChk1*, *PmCDC2*, and *PmCyclin B* was ascertained after dsRNA-Chk1 injection, to examine their relationship with each other. The localization and level of *PmChk1* expression in the hepatopancreas was studied through *in situ* hybridization, and the obtained results were in accordance with those of qRT-PCR. Chk1 activity after dsRNA-Chk1 injection was significantly lower compared with the control group. The GSI of *P*. *monodon* after dsRNA-Chk1 injection was significantly higher than that after injection of dsRNA-GFP or PBS. The present study improves the understanding of the molecular mechanisms underpinning ovarian development in *P*. *monodon*.

## Supporting information

S1 File Sequence of *PmChk1*. (DOCX)
