# Supplementary material for: Genomic structure, expression, and functional characterization of checkpoint kinase 1 from Penaeus monodon
Source: PLoS One. 2018 May 24;13(5):e0198036. doi: 10.1371/journal.pone.0198036 (PMC5967826; doi:10.1371/journal.pone.0198036)
Supplement: S1 File — (DOCX) [file pone.0198036.s001.docx]

AGCGCAGACGCCCTTTTGTAGGTGAGGAGGGCGGGCGGGCGGCGCGGGAGCCCCTTTCGCGTTCGAATTC

GCGGGCGAGCGTAGGCCTAGGGCGCCCCACGTGCTCGGCGGCTGCTCGGCGCCGCAGTCCGCCTCTTTGC

GCTCTATTTCTGGCCATCTGCTCCTCCCGCGGCCGCGAGCAGCAGCCGGGCGGGCGGCGAGGCGAGAGGT

TATGTTGGCTAGAAAGTGTCAGAAAAGACCAGCACCACCATGGCTGGGCCGGTCACCGAATTTGTGGTGG

GGTGGAACGTAGTCCAGACCCTTGGGGAGGGGGCCTTTGGAGAAGTAAAATTGCTAATCAATAAGGACAC

TGGGGAGGCAGTTGCCATGAAGATGGTGGACTTGGTCAAACATCCAGATGCAGCAGATGCTGTGCGCAAG

GAAATATGTCTGCACCGCATGTTAAAACATGCAAACATCATAAAATTTTATGGAAGTCGCCGTGAAAATT

CAATGCAGTACATGTTCCTGGAATATGCTGCAGGTGGTGAACTATTTGATCGTATTGAGCCTGACACGGG

CATGCCTCCCCATCAGGCACAGAAGTACTTCAGGGAATTGATCAGTGGTGTGGAATACCTCCATGGACGG

GGTGTTACACACCGGGATCTCAAGCCAGAGAACTTGCTGCTTGACGAGAATGACCATCTTAAAATAACAG

ACTTTGGAATGGCCACTCTCTTCAGACACAATGGGAAGGAACGTGAATTGGATCGTCGTTGTGGAACAAA

ACCATATATGGCTCCTGAAGTATTGCTGAGACCCTATAATGCTGAACCTGCAGATATTTGGTCTTGTGGA

GTGATTCTTGTTGCACTGCTAGCTGGTGAATTGCCATGGGATGAACCAACTTTTTCTTGTCCAGAATACA

CGGCCTGGAAAGACCGAGATTGTAGGTTGTTCACAACCACACCTTGGACAAAGGTGGACAATCTGGCCCT

CTCTCTTTTGCGCAAGGTATTGAATACTGTACCTAGGCATCGAGCAACAGTACCTCAGGTGAAAGCACAT

CAGTGGTTCACAAAGAACTACCACAAATCTTCAGGTTTTGGACGCAGTGCATCGAATGACAGCATGACCC

CTACTACCAAGCGTGTGTGCAGTGAGCTTGAACGGGAAAACTCCTCATTCTGTTTGGAAGACATGTCAGC

ACGGTTAGCATGTTCACAGCCAGAGGCCCCTACATCTGCTTCAATTAATACTGTTAATGGTCCTAATGTA

GACATGGGGGTTGTGAGCTTCTCGCAACCAGCCCAACCTGACCAGCTGTTGCTCTCCTCTCAACTTACTC

AAAGTACACAAGCAAGTCAAACACCACTGCAGAGGCTTGTGAAACGCATGACTCGTTTGCTTGTAAGGAC

CAACCTGGAGGACACCCTAACTCACCTGGAAGCATTGTTCAACAAGATGAACTACACTTATCGTATGCAC

AATGTCAATGTTCTTACTGTAACCACTCTGGATCGACGTGGAGCTCAACTTGTGCTGAAAGCAAGCATAC

TGGATATGGGCCAGCATATTCTTGTGGATTTCAGACTTTCAAAAGGATGTGGATTGGATTTTAAACGACA

TTTTCTGCGAATTAAAGAGGGTTTATCTCATATAGTAATCAAGGGTCCTGTAACTTGGAATATGGCTCTA

GCAACAAATATGCTACCAGCCTAATTTGACCCAGACAAAATTTTTTGAAGTCTTTCAAAATTTATATGCT

GACTGTTACTCATGCTTGTGTAAGATGTAAAGTATTAGTCTTTAATGAATAAGCTATGATTTATACTCGG

TTAAAGTATAAAAGTGTTTGATATTGAATGTGTGATTAACTTTGAAAGAATTGGATGTAAGACTGGTATG

CCTGTAATAATTTTTCCCTGTCTAGATTAAGCATTTGGTTAACTTGAAAGGTGACCTCCCAGTAATAGTA

GTATGGCAGAATGTTCCAGCAACATTGGCTGCCTGGATAGGTAGTGAGTACCCATCTCAACAGTGTGCTG

CGGTTGTAAGAGAAGTCTTGCTTTTGACCAGCCTTCTCTCTACTCTCGCAGGCAGAATTCTAGTCTTTCA

CTGATGGGTGCTTACTTGCTTTTACGAAAGTAAAAAAAAAAAAAATTGTTCAATCATTCCATTTTCCAAT

TGAGGAAGAAAAACTGTTAACTTACTGTCAGTTAAAGAAATAATTTTTATTTTTATTTACAAGACTTTTA

TTACTCCTATTTTTTGATGGCATATTTCAATTCTCGTGTAGTATAAATAAAGTGATTAAACTGTTCTTAG

TTTATTTCAGTTGAATAAATTTGATTGAAGGATTATCGTGCAAACATTTGGGAGTAGAACTATAAATACC

TATCCTCTAGCTTGTGTAGAGAGAGGGCATGTACTAAGCTTTGGCCCTGTCCACATGAGGGCCAGTTAAC

GCATGGGCACACATCAGAAAGCATGGGCAGTTTCTTGAAATAGCCATGCCTGTTCCCTGCAAGTGGATCT

TTCCAGAGTTAAGAGAATGCGAGGTTGCTATCTCACTTCTGACATGCCAATACCCTTGTGCCAGCGCGGG

CATTCCTTCGTGTGTATAGGGCCCAGGTAGAAATTTATTAGTGTGGTTTCATTGTTTAAAGTGTTGGTAA

AATTTTCACCAACTTTACTTATTTCGTTATTGATGATATGATTAAGTTGAATTGAAAACTTCTGATTAGT

GTAGCATGAAATATTCGATAAGTACATAAAACGGTTTCATCAACCATTGCTGAAGTTTTTGCAACTAGAG

TAGAAATGATGTACATAACATAGTACAAAACTGGATAAAAAATAACGGAATTGACTTTTAAGAATGAAAT

TACTAGTAACTTAAATACAGAAGTACTTCTGTATTCAACTAAAATTTAAAGTATACAAATTGTAAATGGA

GCAAAAGTGAATACAAAAGGTATCCAGAAAATTGTACCTGAAGTGTAAGACCTTTCGAGATGATTCTTGC

ATTGAAGTAATCAGCTAATGGTGTTGACCCCAGAAAACTGCATGATCAATCTAATGATGCACTACAGTCG

AGCATTAACATTTTGATCAATTGTATGAAGATGCTTATTCAGATCCTTTTCCTTGAAGCAAATTCATCTC

GAATTACCCCTTGGCCTTGGCTGTATACTGGCCGATTATTTCCTGTAACCTGAAGAATGTGCAAAATTGA

GTTTTTATGAAGTTGTTACGTTACTTGATTTTGAAGATGTAATTTGCTTCGTATACCCCAGTTATGCTCT

CGGTGATTGTCAAGGAAAAAAAAAAAAAAAAAAAAAAAAAAAAA
